# Dendritic cells enhance the antigen sensitivity of T cells

**DOI:** 10.3389/fimmu.2012.00389

**Published:** 2012-12-26

**Authors:** Natalio Garbi, Thomas Kreutzberg

**Affiliations:** Department of Molecular Immunology, Institutes of Molecular Medicine and Experimental Immunology, University of BonnBonn, Germany

**Keywords:** dendritic cells, T cells, tonic TCR signaling, self-MHC, antigen sensitivity

## Abstract

Naive T cells continuously migrate between the circulatory system and lymphoid organs, where they make dynamic contacts with rare dendritic cells (DCs) that strategically form an extensive dendrite network. In such a scenario, T cells spend most of their time quickly scanning the antigenic content of multiple DCs. These interactions provide the basis for efficient adaptive responses by increasing the probability of encounters between rare antigen-specific T cells and those DCs presenting the respective cognate antigens. In the absence of foreign antigen, however, T cells show different degrees of functional sensitivity toward TCR stimulation. Scanning of MHC/self-peptide complexes by naive T cells in the absence of infection is not without consequences but it increases their subsequent response toward antigenic challenge. This indicates that TCR sensitivity in naive T cells is tuned depending on the MHC/self-peptide signals they integrate from the environment even before T cells encounter cognate antigen. DCs have emerged as key components in providing MHC/self-peptide complexes and increasing the sensitivity of T cells toward subsequent TCR triggering. In the absence of cognate antigen, DCs maintain a tonic TCR signaling and license T cells for immune synapse (IS) maturation resulting in enhanced T cell responses toward a subsequent antigen stimulation. This review discusses recent findings on this subject and highlights the importance of the DC pool size for optimal T cell awareness to foreign antigen.

## INTRODUCTION

Bone marrow (BM)-derived T cell precursors seed the thymus, where they differentiate into mature T cells. During this process, those T cells bearing appropriate α/β T cell receptors (TCR) are positively selected in order to ensure MHC restriction. In addition, those harmful T cells recognizing MHC/self-peptide complexes (referred to as self-MHC hereafter) with high affinity are mostly purged from the repertoire by negative selection. This process of T cell selection in the thymus warrants that peripheral T cells are MHC restricted, so they are able to recognize infected cells, but react only weakly to self-MHC and thus autoimmunity is minimized (Klein et al., 2009).

Once naive T cells exit the thymus, they recirculate between secondary lymphoid organs via blood or lymph. Upon encounter of cognate antigen presented by MHC on dendritic cells (DCs), T cells are primed and differentiate into potent effector cells with the ability to leave the systemic circulation and infiltrate inflamed sites. After the threat is resolved, memory CD4 and CD8 T cells remain patrolling the body and act as sentinels for fast responses against secondary infections. Different memory T cell populations have been described, some of them recirculating between lymphoid organs and others being present at peripheral tissues where the initial infection took place (Sallusto et al., 2004; Harty and Badovinac, 2008; Wakim and Bevan, 2010; Jiang et al., 2012). Therefore, naive, effector, and memory T cells all encounter antigen-presenting cells (APCs) such as DCs in different environments, i.e., lymphoid and non-lymphoid organs.

Dendritic cells are BM-derived APCs that are crucial for initiating T cell responses (Steinman and Cohn, 1974; Jung et al., 2002). One of their hallmarks is to excel in antigen presentation on MHC-I and -II to CD8 and CD4 T cells, respectively (signal 1), provide costimulatory signals (signal 2), and promote the differentiation of naive T cells into specialized effector cells via the provision of key cytokines (signal 3; Sporri and Reis e Sousa, 2005; Heath and Carbone, 2009; Joffre et al., 2009; Segura and Villadangos, 2009; Kurts et al., 2010). It is commonly accepted that the capacity of DCs to provide signals 1, 2, and 3 simultaneously makes them specially suited to promote priming of naive T cells. In addition, DCs are located in the T cell areas of lymphoid organs, or easily migrate into them upon activation, forming an extensive network of dendrites thus providing a topographical context in which DCs and T cells interact (Lindquist et al., 2004). This may be an important differential feature of DCs, since other professional APCs such as B cells also express high levels of costimulatory molecules and produce a variety of T cell growth factors, but are not located in the T cell area under normal conditions.

During the steady state, T cells frequently contact DCs in secondary lymphoid organs. There are at least two important consequences of these frequent contacts: (1) they increase the likelihood for encounters between extremely low frequencies of antigen-specific naive T cells and the few DCs presenting the respective cognate antigen; (2) self-MHC recognition on DCs in the absence of cognate antigen induces a basal, tonic TCR signaling that augments the antigen sensitivity of T cells (**Box 1**). This review focuses on recent developments by which self-MHC recognition on DCs prior to an encounter with foreign antigen induces tonic TCR signaling thereby increases the awareness of T cells for subsequent encounters with their cognate antigen. Finally, we discuss some key questions in this field that remain to be answered.

**Box 1. Summary of self-MHC recognition, tonic TCR signaling and antigen sensitivity.**

*Antigen sensitivity* is the capacity of T cells to respond to TCR stimulation via cognate MHC/antigen recognition to become activated and undergo proliferation. The higher the sensitivity, the lower the amount of MHC/antigen recognition required to trigger full T cell activation. T cells can undergo different states of antigen sensitivity depending on the cues they integrate from the environment. A key cue is the recognition of *MHC/self-peptide complexes* (referred to as self-MHC), which induces a basal level of TCR activation resulting in increased sensitivity toward cognate antigen (Stefanova et al., 2003; Hochweller et al., 2010). This basal activation of the TCR complex is also referred to as *tonic TCR signaling* and is exemplified by low levels of CD3ξ phosphorylation. Thus, self-MHC recognition increases the awareness of T cells and licenses them to respond to lower amounts of cognate antigen.

When does self-MHC recognition increase the antigen sensitivity of T cells? There are two stages during which self-MHC recognition increases the T cell antigen sensitivity: *prior to* and *concomitant to* recognition of foreign antigen:

*Self-MHC recognition in the absence of cognate antigen*. DCs and T cells continuously interact in secondary lymphoid organs. Self-MHC recognition by T cells results in tonic TCR signaling and increased T cell responsiveness toward a subsequent encounter with cognate antigen. The nature of the self-peptide(s) is presently unknown.

*Concomitant recognition of self- and foreign-antigen bound to MHC*. The sole recognition of MHC/foreign-peptide complexes is inefficient to trigger naive T cell activation. Co-recognition of self-MHC complexes dramatically increases T cell responsiveness (Krogsgaard et al., 2005). The same self-MHC complexes that drive positive selection in the thymus have been shown to increase the antigen sensitivity during concomitant recognition of foreign antigen (Ebert et al., 2009; Lo et al., 2009).

## T CELLS FREQUENTLY CONTACT DCs IN SECONDARY LYMPHOID ORGANS IN THE STEADY STATE

The frequent contacts between T cells and DCs provide a structural basis for the uniqueness of DCs in T cell priming. Elegant *in vivo* two-photon microscopy experiments have provided important insights into the kinetics of T cell priming (for reviews, see Bousso and Robey, 2003; von Andrian and Mempel, 2003; Cahalan and Parker, 2005; Cahalan and Gutman, 2006; Germain et al., 2008; Kastenmuller et al., 2010).

In the absence of cognate antigen, T cells and DCs move along networks of reticular fibroblasts (Bajenoff et al., 2006), with T cell motility appearing to be otherwise random (Miller et al., 2002, 2004a; Textor et al., 2011). The average speed of naive CD4 and CD8 T cells in the absence of antigen has been reported to vary between about 6 μm/min (Skokos et al., 2007) and 18 μm/min (Textor et al., 2011), with most reports showing an average speed of about 10 μm/min (Miller et al., 2002, 2004a; Bousso and Robey, 2003; Hugues et al., 2004; Mempel et al., 2004; Shakhar et al., 2005). These variations may likely be due to differences in the T cell clonality, technical issues, as well as the depth of imaging in the lymph node (LN) which has been shown to significantly impact T cell speed (Worbs et al., 2007). In the absence of cognate antigen, it has been estimated that the mean transit time in LNs is about 10 h for CD4 T cells and about 20 h for CD8 T cells, with considerable variation depending on the particular LN. Of this time, about one-third is spent interacting with MHC molecules on DCs (Mandl et al., 2012), with the majority of the contacts between T cells and DCs lasting between 3 and 5 min (Miller et al., 2004a,b; Mandl et al., 2012). These interactions are highly dynamic, as CD4 T cells undergo 160–200 contacts with DCs during their transit time in the LNs, whereas CD8 T cells undergo about 300 contacts (Mandl et al., 2012). On the other side, each DC is contacted by about 500 CD8 T cells (Bousso and Robey, 2003) or 5000 CD4 T cells (Miller et al., 2004a) per hour. Thus, T cells frequently scan the surface of DCs during their transit through secondary lymphoid organs in the absence of foreign antigen. It is generally accepted that these frequent contacts serve as a “finding needle in the haystack” function: otherwise impossible interactions can proceed between extremely rare antigen-specific T cells and DCs presenting that particular antigen. Regarding the kinetics of T cell priming, different laboratories using intravital two-photon microscopy have reached similar conclusions: following recognition of cognate antigen on DCs, T cells undergo activation in three distinct and sequential phases (Hugues et al., 2004; Mempel et al., 2004; Miller et al., 2004a,b). Within 8 h of access to the T cell zone, T cells slow down their mean speed to about 4 μm/min in average engaging serial encounters with DCs bearing cognate antigen. By sampling the antigen dose during this initial phase, T cells become activated and make the decision of whether they enter the next phase of T cell activation (Henrickson et al., 2008). This phase is characterized by taking place over a longer period of time (about 12 h) in which an arrest of T cell mobility is observed with prolonged interactions with DCs, which may last longer than 1 h. Consequently, T cell speed is halved during this phase. Coinciding with T cell proliferation, T cells disengage from these stable contacts with DCs and enter the final phase of T cell activation by resuming their motile behavior serially interacting with different DCs. Integration of signals derived from serial encounters with DCs bearing cognate antigen has been shown to increase the effector function of T cells (Celli et al., 2005). Following activation of CD4 T cells, naive CD8 T cells undergo directional rather than random migration toward DC–CD4 T cell conjugates via a CCL3 and CCL4 gradient, thereby increasing the likelihood of receiving help to increase their cytolytic and recall responses (Castellino et al., 2006). A similar process has been observed during alternative cross-priming whereby activated NKT cells attract naive CD8 T cells to the relevant DCs via CCL17 (Semmling et al., 2010). In summary, T cells frequently sample the surface of DCs in a highly dynamic fashion during their transit through secondary LNs in the absence of cognate antigen. Upon encounter with cognate antigen, T cells change their kinetic behavior and undergo intense interactions with antigen-bearing DCs.

Besides increasing the likelihood for T cells finding the DC presenting the respective cognate antigen, the frequent interactions between DCs and T cells in the steady state (absence of cognate antigen) have in addition two other major consequences: (1) recognition of self-antigen on DCs outside the thymus results in peripheral tolerance, i.e., deletion of self-reactive T cells thereby minimizing autoimmunity (Kurts et al., 1997, 2001; Probst et al., 2003), and (2) recognition of self-MHC on DCs induces a tonic TCR signaling that promotes the sensitivity of T cells toward their cognate antigen (Stefanova et al., 2002; Hochweller et al., 2010). These two major functions of steady-state DCs seem at first contradictory. We have proposed that the affinity of TCR/self-MHC recognition dictates the final T cell outcome: high-affinity interactions lead to T cell deletion, whereas those of weaker affinity promote T cell antigen sensitivity (Garbi et al., 2010).

## DENDRITIC CELLS ARE REQUIRED TO MAINTAIN THE ANTIGEN SENSITIVITY OF NAIVE T CELLS

**FIGURE 1:**
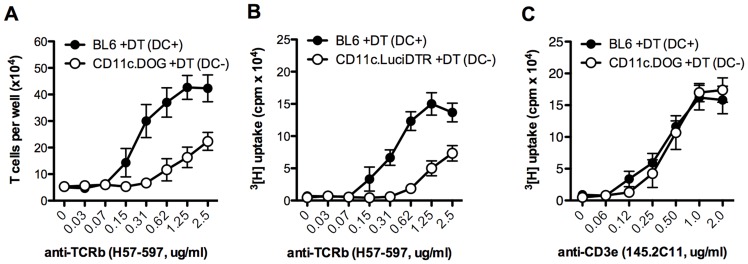
**CD4 T cell proliferation in mice lacking DCs**. 5 × 10^4^ MACS-purified CD4 T cells from the indicated mouse strain were activated for 4 days with the specified concentration of plate-bound anti-TCRβ (clone H57-597) or anti-CD3ε (145.2C11) antibodies. Proliferation was quantified either by the number of T cells per well at the end of the experiment **(A)**, or by incorporation of ^3^[H]-thymidine for the last 9 h of the experiment **(B,C)**. Results are expressed as mean ± SEM (*n* = 3 mice). Shown is one representative of three experiments. Similar results were obtained with BL6 mice treated with DT or with the respective transgenic mice treated with PBS.

By analyzing CD5 expression as a surrogate marker of TCR triggering, it has recently been shown that most of the tonic TCR signaling of naive T cells occurs in the secondary lymphoid organs (Mandl et al., 2012). Most contacts between T cells and DCs in the LN take place for about 5 min. For CD4 T cells, these contacts are highly dependent on MHC-II expression by the DC, because absence of MHC-II results in shorter interactions of about 2 min (Mandl et al., 2012). Pioneering work in Germain’s laboratory showed that self-MHC recognition by T cells in the absence of cognate antigen resulted in basal activation of the TCR complex and increased antigen sensitivity of T cells toward subsequent encounters with their cognate antigen (Stefanova et al., 2002). The requirement of DCs for tonic TCR signaling and maintenance of the antigen sensitivity in T cells was described in transgenic CD11c.DOG mice, in which DCs express the human diphtheria toxin receptor (DTR) and thus can be depleted by single or repetitive administrations of diphtheria toxin (DT; Hochweller et al., 2008). In these mice, naive CD4 and CD8 T cells isolated after DT application show a marked hypoproliferative response against a variety of antigens presented by professional APCs, including cognate peptide, superantigen (Hochweller et al., 2010), and anti-TCRβ antibody (**Figure 1A**). These results indicate that DC–T cell interactions in the steady state in the absence of cognate antigen are required to maintain the sensitivity of naive T cells for their cognate antigen. Similar results have been obtained in other transgenic mouse strains such as CD11c.DTR (Hochweller et al., 2010), and the recently described CD11c.LuciDTR (**Figure 1B**) that expresses luciferase and DTR under the CD11c promoter (Tittel et al., 2012). The proliferative response to anti-CD3ε antibody is, however, not compromised in T cells from DC-depleted mice (Birnberg et al., 2008; **Figure 1C**). Although at present we cannot explain why T cells from DC-depleted mice are able to respond normally to anti-CD3ε stimulation, but not to activation with MHC/antigen or anti-TCRβ antibody, differences in the binding affinities or in the ability of anti-TCRβ and anti-CD3ε antibodies to cross-link different TCR complexes may contribute to explain this paradox.

Tuning of the T cell antigen sensitivity is a dynamic process that depends on fast interactions with DCs. Antigen sensitivity is lost very quickly after disruption of cell–cell contacts (within 15 min; Stefanova et al., 2002), and it is regained also very promptly, within 30 min of reintroduced DC–T cell contacts (Hochweller et al., 2010). The loss of antigen sensitivity is not associated to decreased viability of T cells following DC depletion. Both the frequency and the numbers of viable T cells is not altered in DC-depleted mice (Hochweller et al., 2010), which is consistent with findings that mice constitutively lacking DCs do not present reduced T cell counts (Birnberg et al., 2008; Ohnmacht et al., 2009).

Studies using DC depletion have demonstrated that DCs are required to maintain the sensitivity of T cells for subsequent challenges with their cognate antigen. Both splenic CD8^+^ and CD8^-^ DCs are equally suited for tuning the T cell’s antigen sensitivity (Hochweller et al., 2010). B cells are also able to maintain T cell responsiveness *in vitro*, although due to anatomical restrictions *in vivo*, naive T cells will only seldomly interact with B cells at the borders between the T and B cell zones. However, not all APCs can do it; macrophages are not able to promote T cell antigen sensitivity (Hochweller et al., 2010), and whether this is due to differences in the expression level of self-MHC or other molecules is still an open question. A central question was how many DCs are required to maintain the T cell sensitivity? Using mixed BM chimeras in which a graded percentage of DCs expressed DTR, we showed that depletion of only half of the DC compartment already resulted in partial loss of antigen sensitivity. The activation status of the remaining DCs was not altered regarding expression of MHC-I and -II, and costimulatory molecules (N. Garbi, unpublished data), suggesting that the level of DC activation does not play a key role in the maintenance of T cell antigen sensitivity. Thus, minor alterations in the size of the DC compartment seem to have an impact on the T cell responsiveness. This is particularly important in view of the rapid turnover of DCs in the lymphoid organs of mice. Depending on the methodology used, the half-life of cDCs in the spleen has been estimated to be between about 2 days (Kamath et al., 2002) and 7 days (Liu et al., 2007), the whole splenic DC compartment is replaced about 150 or 45 times, respectively, during the lifespan of a laboratory mouse.

## DEPENDENCE ON SELF-MHC RECOGNITION AND COSTIMULATORY MOLECULES

Seminal work by the group of Germain, demonstrated that recognition of self-MHC class II by CD4 T cells promoted the sensitivity of naive CD4 T cells against a subsequent challenge with cognate antigen (Stefanova et al., 2002, 2003). This work demonstrated that in the steady state, the TCR is actively integrating cues from self-MHC recognition leading to a basal activation of proximal TCR signaling events, specifically CD3z phosphorylation (Stefanova et al., 2002). A subsequent study showed that self-MHC-II recognition was required to promote CD4 T cell antigen sensitivity also *in vivo* (Fischer et al., 2007). DCs were later identified as the cells providing self-MHC recognition to CD4 and CD8 T cells resulting in increased T cell antigen sensitivity toward subsequent challenges (Garbi et al., 2010; Hochweller et al., 2010). Therefore, the requirement of DCs to maintain T cell antigen sensitivity is molecularly based on recognition of self-MHC. Indeed, interaction of DCs and T cells resulted in a specific increase in the basal phosphorylation of ZAP70-associated CD3ξ, although the total levels of ZAP70 and CD3ξ were not altered (Hochweller et al., 2010). Thus, self-MHC recognition on DCs induces tonic TCR signaling that is critical to maintain antigen sensitivity. It is presently unclear whether more upstream events in TCR signaling are also affected by lack of DC–T cell interaction, for instance recruitment of the NCK adaptors into the TCR complex, which is known to modulate the TCR antigen sensitivity (Roy et al., 2010).

The loss in antigen sensitivity is not accompanied by changes in the expression of molecules known to modulate TCR signaling such as TCRβ, CD3ε, CD3ξ, CD4, and CD8α (Hochweller et al., 2010), or in global gene expression (Hochweller and Garbi, unpublished data), suggesting specific defects in signaling events rather than in expression patterns. This is supported by the rapid loss of antigen sensitivity after disruption of DC–T cell contacts (~15 min; Hochweller et al., 2010) and rapid reconstitution upon contact reintroduction (~30 min; Stefanova et al., 2002). In addition, T cells from DC-depleted mice proliferate normally in response to TCR-independent stimuli such as ConA or PMA/ionomycin stimulation, indicating that they do not have a global defect in cell cycle entry.

The requirement for self-MHC recognition on DCs to promote T cell responsiveness toward a subsequent antigenic challenge is reminiscent of recent data showing that self-MHC-II recognition at the time of foreign antigen recognition also increases the response of CD4 T cells to their cognate antigen (Ebert et al., 2009; Lo et al., 2009) in what has been defined as the pseudodimer model (Krogsgaard et al., 2005, 2007). The nature of the MHC class I/peptide complex required to maintain CD8 T cell antigen sensitivity is less clear. Our results show that CD8 T cells require prior self-MHC recognition on DCs to maximally respond to a subsequent antigenic challenge (Hochweller et al., 2010). In analogy to the pseudodimer model for CD4 T cell activation, simultaneous recognition of MHC class I molecules loaded with foreign stimulating peptide and with endogenous non-stimulating peptides strongly increases the sensitivity to the former (Purbhoo et al., 2004; Cebecauer et al., 2005; Yachi et al., 2005, 2007; Anikeeva et al., 2006). However, as opposed to CD4 T cells, all tested MHC class I-binding peptides served as coagonists (Yachi et al., 2005, 2007), suggesting that it is the interaction between CD8 coreceptor and MHC class I/endogenous peptide what is required to amplify responses against cognate antigens and not the specific TCR-MHC/self peptide recognition observed for CD4 T cells (Gascoigne, 2008). This hypothesis is supported by the finding that the CD8 coreceptor, but not CD4 is required to increase sensitivity of T cells at high density of peptide ligands (Purbhoo et al., 2004). However, as for the maintenance of CD4 T cell antigen sensitivity, it remains unknown whether specific MHC class I/endogenous peptide complexes need to be recognized prior to foreign antigen challenge for maximal responses.

Altered peptide ligands (APLs) bound to MHC have been shown to partially activate the TCR complex (Evavold et al., 1993). However, the outcome of these partial TCR activation dramatically differs from the tonic TCR signaling induced by self-MHC recognition discussed in this review. APLs often result in (1) partial T cell activation leading to functional T cell anergy in response to subsequent encounter with cognate antigen, or (2) TCR antagonism when recognized simultaneously with cognate antigen (Sloan-Lancaster and Allen, 1996). Although some endogenous self-peptides have been shown to function as APL for a given TCR clone (Evavold et al., 1995), self-MHC ligands inducing tonic signaling do not induce T cell activation (as defined by the “quiescent” state of naive T cells *in vivo*) but increase their sensitivity toward subsequent encounters with cognate antigens. Although presently unknown, the biochemical basis for the difference between self-ligands inducing T cell anergy (APLs) and those inducing productive tonic TCR signaling may reside in the affinity for the TCR.

Thus, self-MHC recognition tunes T cell responsiveness toward foreign antigen in two different contexts: first, exclusive self-MHC recognition in the absence of foreign antigen results in tonic TCR signaling and enhanced T cell responsiveness to a subsequent challenge with cognate antigen; second, as defined in the pseudodimer model, concomitant recognition of MHC molecules loaded with self- and foreign- peptides leads to increased sensitivity to the later.

Interestingly, it is the same ligands driving positive selection in the thymus that increase the CD4 T cell responsiveness toward cognate antigen when recognized simultaneously in the periphery (Ebert et al., 2009; Lo et al., 2009). We proposed that a similar mechanism is in place to promote responsiveness to subsequent antigenic challenge, i.e., it is the recognition in the periphery of the ligands inducing positive selection in the thymus that results in tonic TCR signaling and increased T cell antigen sensitivity (Garbi et al., 2010). Although this hypothesis is not formally proven yet, Stefanova et al. (2002) demonstrated that recognition of the same MHC class II restriction element that drives positive selection of AND TCR transgenic CD4 T cells is required to maintain their antigen responsiveness in the periphery. Whether this finding can be generalized to other TCR specificities is still an open issue, but strongly suggests that the selecting MHC class II haplotype is required and that the mere interaction between MHC-II and the CD4 coreceptor is not sufficient to maintain antigen sensitivity (Stefanova et al., 2002).

Presently, it is still unclear whether other molecular cues between DCs and T cells participate in promoting antigen sensitivity in addition to self-MHC recognition. MHC-deficient DCs are able to partially maintain T cell responsiveness, albeit to a much lower degree than their MHC-sufficient counterparts (Hochweller et al., 2010). DCs express large amounts of costimulatory molecules such as CD80 and CD86 in the steady state. Because activation of their receptor CD28 synergizes TCR engagement of cognate antigen to bolster T cell proliferation, it is tempting to speculate that CD28 ligation may also synergize with self-MHC recognition to promote tonic TCR signaling. In addition, other mechanisms may also be involved. In this context, non-MHC-dependent contact of T cells to DCs induces a transient semi-activation of the former resulting in enhanced T cell responses to subsequent cognate antigen in a process known as “adhesion-induced T cell priming” (Revy et al., 2001). However, this phenomenon is not specific to interaction with DCs because adhesion to other cell types, immobilized ligands or even glass had a similar effect (Randriamampita et al., 2003).

## ARE DENDRITIC CELLS REQUIRED TO MAINTAIN THE ANTIGEN SENSITIVITY OF OTHER T CELL POPULATIONS: EFFECTOR, MEMORY, AND REGULATORY T CELLS?

Presently it is unknown whether effector or memory T cells in the steady state are dependent on DC-induced tonic TCR signaling to increase their sensitivity against a subsequent challenge with cognate antigen. During infection, memory CD8 T cells interact with DCs in lymphoid and non-lymphoid sites resulting in antigen-specific reactivation (Belz et al., 2007; Wakim et al., 2008). However, further experiments are needed to determine whether effector/memory T cells also depend on constant self-MHC recognition on DCs in the absence of infection to increase their sensitivity against a subsequent antigen encounter.

In the different context of simultaneous recognition of self- and cognate-antigen, effector T cells seem to be less dependent on self-MHC recognition than their naive counterparts for antigen-specific responses (Yachi et al., 2007). Based on those findings, we hypothesize that effector/memory T cells are also less dependent on recognition of self-MHC in the steady state to increase their sensitivity to further cognate antigenic challenge.

**FIGURE 2:**
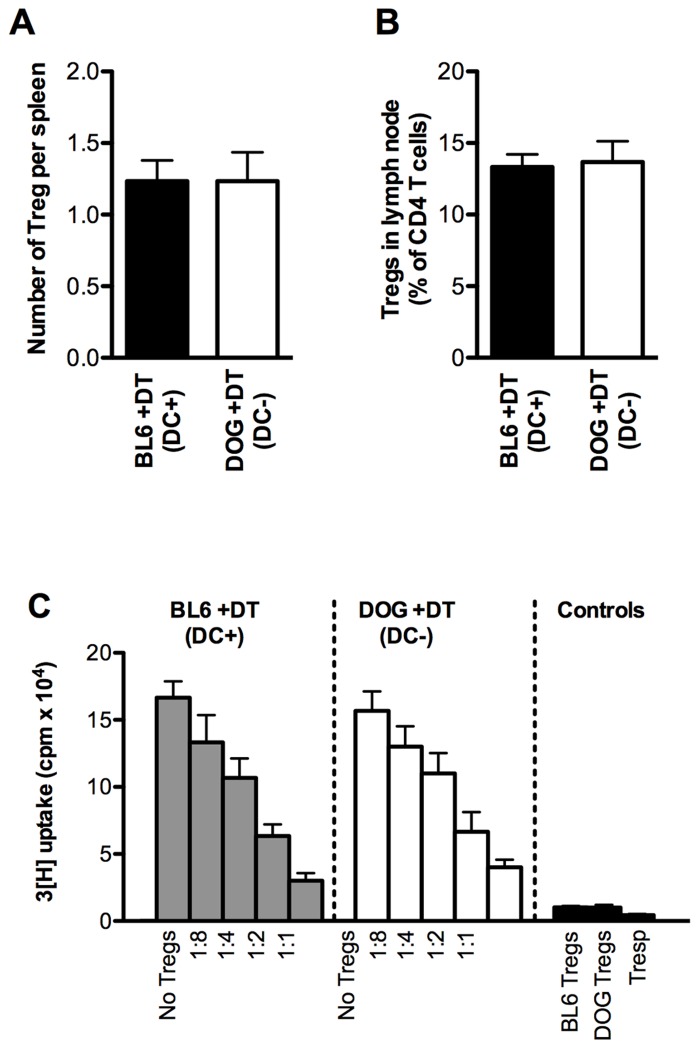
**Treg numbers and function from mice lacking DCs**. **(A)** Absolute numbers of Treg cells in the spleen of mice with (DC^+^) or without (DC^-^) DCs 2 days after DT administration. Tregs were identified as live CD4^+^ FoxP3^+^ lymphocytes by flow cytometry. **(B)** Frequency of Tregs in skin-draining lymph nodes of mice with (DC^+^) or without (DC^-^) DCs two days after DT administration. **(C)** Standard *in vitro* assay for suppressive capacity of Treg cells isolated from the spleen of mice with (DC^+^; gray bars) or without (DC^-^; white bars) DCs 2 days after DT administration. Live CD4^+^CD25^high^ Tregs were pooled from three mice and sorted using flow cytometry. Live CD4^+^CD25^-^ responder T cells were sorted using flow cytometry from the spleen of untreated BL6 mice. Responder T cells were stimulated in the presence of irradiated APCs, anti-CD3ε antibody (clone 145.2C11, 2 μg/ml) and titrated amounts of Treg cells for 4 days. Proliferation of responder cells was assayed by ^3^[H]-thymidine for the last 9 h of the experiment. Results are expressed as mean ± SEM. For **(A,B)**, *n* = 3 mice; For **(C)**, *n* = 3 wells. Shown is one representative of three experiments.

There is some correlative evidence that DCs regulate the size of the Treg compartment in a positive manner. In mice depleted for DCs or lacking DCs constitutively, a reduced frequency of Tregs by a factor of approximately 2–3 has been reported in the spleen, LNs, and/or blood (Darrasse-Jeze et al., 2009; Bar-On et al., 2011). However, in other reports, no differences or very small differences in the number of Tregs in the spleen and/or LNs were reported in mice constitutively lacking DCs (Birnberg et al., 2008; Ohnmacht et al., 2009). In addition, DC depletion did not result in decreased suppressive function of splenic Treg cells (Birnberg et al., 2008). Following depletion of DCs for 2 days in CD11c.DOG, we did not observe any alteration in the number of Treg cells, suppressive capacity or phenotype in the spleen (**Figure 2** and unpublished data). Our results and those by Birnberg et al. suggest that DCs are not required to maintain the suppressive capacity of Tregs. Therefore, further studies are required to investigate the apparently contradictory results on the role of DCs in the maintenance of Treg homeostasis.

## DENDRITIC CELLS LICENSE T CELLS FOR IMMUNE SYNAPSE FORMATION

Following TCR signaling in response to recognition of foreign antigen, T cell surface molecules and scaffolding protein are redistributed and enriched in the contact zone between T cells and APCs, resulting in the generation and maturation of the IS. The IS is characterized by a central enrichment of TCR and CD3 molecules termed central supramolecular activation cluster (cSMAC) that is surrounded by a further cluster formed by LFA-1, also called peripheral SMAC (Grakoui et al., 1999). Initial TCR triggering results in the so-called inside-out signaling leading to activation of LFA-1 (Kinashi, 2005). In turn, activated LFA-1 binds to ICAM-1 molecules on the APC promoting firm T cell-APC adhesion (Lim et al., 2011) and further TCR/CD3 signaling events (Davis and Dustin, 2004; Fooksman et al., 2010).

Naive CD4 T cells isolated from DC-depleted mice fail at developing a mature IS following recognition of their cognate antigen (Hochweller et al., 2010), indicating that the tonic TCR signaling resulting from self-MHC recognition is also required for licensing T cells for IS maturation. The key question here is whether an impaired IS maturation is the consequence or the reason for defective TCR signaling and T cell proliferation. In other words, do hyporesponsive T cells fail to mount a mature synapse due to defective inside-out signaling resulting in impaired TCR signal transduction and proliferation?, or is the TCR signaling cascade itself defective and, consequently, there is lack of LFA-1 activation and IS formation? These questions remain to be elucidated yet.

## MODEL OF LOCATION-DEPENDENT T CELL ANTIGEN SENSITIVITY

As discussed earlier, the antigen sensitivity of naive T cells is continuously fine-tuned depending on whether or not T cells interact with DCs. Self-MHC recognition on DCs results in a rapid increase in the sensitivity of the TCR for a subsequent antigenic challenge, whereas lack of self-MHC recognition leads to a rapid loss of sensitivity (Stefanova et al., 2002; Hochweller et al., 2010). Both of these processes take place within minutes following initiation or disruption of DC–T cell interaction, thus the loss of T cell responsiveness to cognate antigen caused by reduced interactions is quickly reverted after reintroduction of DC–T cell contacts. Naive T cells continuously recirculate between lymphoid organs and the systemic circulation where they spend only about 30 min (Pabst, 1988). In the blood, where self-MHC recognition on DCs is very unlikely, CD4 T cells show reduced tonic TCR signaling and responsiveness to TCR stimulation (Stefanova et al., 2002). Consequently, it has been shown recently that most of the tonic TCR signaling in the steady state takes place within the secondary lymphoid organs (Mandl et al., 2012). It is therefore crucial that naive T cells recover quickly their TCR responsiveness upon re-entering lymphoid organs and interacting with DCs to ensure optimal responses against foreign antigens. Indeed, the state of T cell hyporesponsiveness is completely reverted 30 min after reintroducing DC–T cell interaction (Hochweller et al., 2010).

Thus, T cells appear to go through several rounds of normal and hyporesponsive states toward cognate antigen depending on their location at a given time: they are fully responsive in the lymphoid organs, where they can be primed against invading antigens, whereas they remain hyporesponsive in the blood where priming is not supported mainly due to anatomical restrictions. Presently, it is difficult to understand the physiological relevance of intermittently loosing TCR antigen sensitivity each time that T cells enter the systemic circulation. It may serve as a transient “metabolic rest” facilitating T cells to increase their tonic TCR signaling and antigen sensitivity upon re-entering lymphoid organs, where they have to be fully aware of minute amounts of foreign antigen displayed by DCs at the initial stages of an infection.

In addition, self-MHC recognition during the steady state also affects other responses mediated by T cells. Recently, Hünig’s group has shown that the proliferative response of human T cells to the superagonist CD28 TGN1412 antibody is also dependent on tonic TCR signaling maintained by MHC scanning (Romer et al., 2011; Hunig, 2012). Similarly, it has been shown that naive CD8 T cells require self-MHC recognition in order to become proliferative in response to IL-2 and IL-15 (Cho et al., 2010). Therefore, self-MHC recognition induces tonic TCR signaling that is required not only for increasing TCR sensitivity to cognate antigen, but also for optimizing responses against other TCR-independent stimuli.

## INHIBITING DENDRITIC CELL APOPTOSIS LEADS TO AN INCREASE IN DENDRITIC CELL FREQUENCY AND T CELL HYPERACTIVATION

**FIGURE 3:**
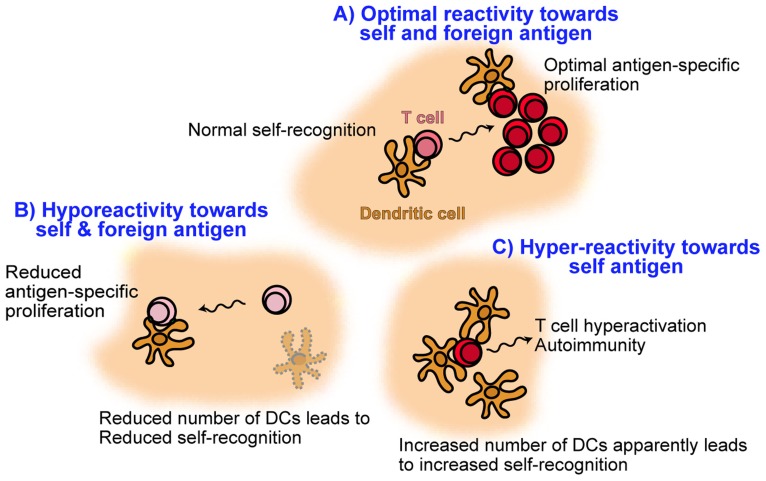
**Model illustrating the role of dendritic cell frequency on promoting healthy T cell responses**. Under normal DC homeostasis **(A)**, naive T cells recognize self-MHC on DCs resulting in tonic TCR cell signaling and increased antigen sensitivity. As a result, subsequent foreign antigen challenge leads to optimal T cell activation and proliferation. However, upon conditions of reduced self-MHC recognition on DCs such as DC depletion **(B)**, T cells undergo reduced tonic TCR signaling and decreased antigen sensitivity. These T cells become hyporesponsive and are not able to undergo strong proliferation in response to antigenic challenge. On the other hand, an increase in the number of DCs **(C)** apparently results in T cell hyperactivation, possibly due to increased self-MHC recognition, and autoimmunity.

Self-MHC recognition on DCs results in enhanced T cell antigen sensitivity and optimal proliferation in response to cognate antigen. As discussed here, a decrease in DC numbers results in hyporesponsive T cells that fail to proliferate to a normal level. Just a twofold decrease in the numbers of DCs already results in partially reduced T cell proliferation (Hochweller et al., 2010). Interestingly, the opposite also seems to apply: an increase of about threefold in the frequency of DCs results in T cell hyperactivation and autoimmunity (Chen et al., 2006). Enforced expression of the baculoviral antiapoptotic p35 protein by DCs, resulted in DC accumulation and chronic T cell hyperactivation leading to multiorgan infiltration and production of autoantibodies (Chen et al., 2006). MHC-II and CD40 expression, hallmarks of DC activation, were unaltered in that study, suggesting that T cell hyperactivation was a result of increased DC frequency rather than activation due to increased half-life. Thus, DC homeostasis in the absence of foreign cognate T cell antigen is critical to ensure optimal T cell responses to subsequent challenges with cognate antigen: whereas too few DCs result in reduced antigen sensitivity of T cells, a sustained increase in the number of DCs apparently leads to T cell hyperactivation and autoimmunity. These findings are summarized in **Figure 3** and highlight the importance in maintaining the correct size of the DC pool to promote healthy T cell responses.

## CONCLUSIONS AND OPEN QUESTIONS

There is mounting evidence that self-MHC recognition in the periphery is critical for several processes including: (i) maintenance of tonic TCR signaling and T cell antigen sensitivity, which are critical for optimal responses to subsequent challenge with cognate antigen; (ii) synergism at the time of cognate antigen recognition leading to increased T cell responses; (iii) increased TCR-independent T cell proliferative responses to various stimuli such as superagonist CD28 TGN1412, IL-2, and IL-15. The former two are mediated by self-MHC recognition on DCs, whereas the role of DCs in providing self-MHC for the TCR-independent responses is not clear yet.

Despite these advances several open questions are remaining. Amongst these, the following are central to understand the molecular mechanisms of DC-induced tonic TCR signaling:

(1) Characterization of the signaling events induced by self-MHC recognition on DCs resulting in increased T cell antigen sensitivity. It is clear that self-MHC recognition induces tonic TCR signaling by partial CD3ξ phosphorylation. The finding that the maturation of the IS is compromised in DC-less T cells, opens the possibility that beyond tonic TCR signaling, integrin (such as LFA-1) activation is impaired following stimulation with cognate antigen, leading to deficient IS maturation and thus reduced T cell proliferative responses.
(2) Are there other molecular events in DC–T cell interactions that contribute to maintenance of the T cell antigen sensitivity? Hypothetically, costimulatory molecules such as CD80 and CD86 may participate in the tonic T cell signaling by partially activating CD28 in the absence of cognate antigen. Costimulation plays a key role in enhancing the proliferative response to TCR stimulation. Whether this also applies to basal TCR signaling promoted by self-MHC recognition is unclear.
(3) What is the nature of the self-MHC ligands required to induce tonic TCR signaling? We have previously proposed that these are the same ligands that induce positive selection in the thymus, but it needs to be demonstrated.
(4) Do memory T cells require tonic TCR signaling for enhanced responses to antigenic rechallenge? Different subtypes of memory T cells reside in lymphoid and extra-lymphoid compartments. DCs have been shown to interact with memory T cells and to be required for maximal T cell restimulation following antigenic rechallenge both in lymphoid organs and in extralymphoid organs (Zammit et al., 2005; Wakim et al., 2008). However, it remains open whether or not memory T cells also require tonic T cell signaling for increased antigen sensitivity.

## Conflict of Interest Statement

The authors declare that the research was conducted in the absence of any commercial or financial relationships that could be construed as a potential conflict of interest.
